# New Adhesive Dressing

**Published:** 1850-11

**Authors:** 


ARTICLE II.

*New Adhesive Dressing* —By Dr. Mellez.

The eagerness with which collodion was adopted by many practitioners shows their want of satisfaction with the materials commonly in use. Certain disadvantages attach to all the ordinary means of keeping wounds in apposition, such as sutures, adhesive and isinglass plasters. Collodion, which possesses many valuable qualities as an adhesive, requires, in order to give satisfactory results, to be of a precise degree of concentration, and, like other dressings, demands, in order to be nicely applied to wounds, the aid of assistants, which is not always at the command of the surgeon, especially in country practice where he must often operate without the aid of others, and where the patients cannot be visited, except at long intervals.

From his dissatisfaction with the ordinary adhesive dressings applied under such circumstances, Dr. Mellez was led to adopt a solution of "gum lac," which is much used in the arts as a varnish and adhesive.

He uses a solution of this substance in spirit of wine, made with the aid of a moderate heat and in such proportion as to give a mixture having the consistence of jelly, or nearly approaching to it. It can be made in a common wide-mouthed bottle, and a simple cork suffices for its preservation. When he uses it, he spreads it with a spatula upon pieces of cloth or taffetas, cut to the size required. According to him, it possesses all the good properties of collodion, and even higher properties than that substance—viz., contraction during the desiccation, impermeability to air, absence of all irritating action on the skin or wound, intimate adherence to the skin, and resistance to the action of water, fatty matters, or the discharges from the wound. It has not, like collodion, the quality of being colorless; but he believes that it might, if required, be decolorized, and, if applied upon animal membrane, would furnish a transparent dressing. It does not dry so quickly as collodion, but that is the only advantage the latter possesses. The lac dressing, however, does not require so long a time as to be inconvenient to the surgeon. Moreover, it is not indispensable for the lac, as it is for the collodion, that the skin be absolutely dry. It has further the advantage (not to be overlooked) of being more moderate in cost.—*Monthly Jour., from Amer. Jour.*

